# Epidemiological trends and burden of gout in China and the European Union: a GBD 2023 and Mendelian randomization study

**DOI:** 10.1007/s10067-026-08135-6

**Published:** 2026-05-05

**Authors:** Xiaolong Lyu, Vahid Jahed, Wenzheng Ding, Xiaolei Sun, Reem Jamous, Junwen Zheng, Zahra Sabouri, Christian Heiss, Houmam Anees, Thaqif El Khassawna

**Affiliations:** 1 Experimental Trauma Surgery, Faculty of Medicine, Justus-Liebig-University of Giessen, 35392 Giessen, Germany; 2 Department of Trauma, Hand and Reconstructive Surgery, Faculty of Medicine, Justus-Liebig-University of Giessen, 35392 Giessen, Germany; 3 Department of Emergency Medicine, Gaomi People’s Hospital, Gaomi, Shandong China; 4 Biruni University, Istanbul, Türkiye; 5 School of Pharmacy, The University of Jordan, Amman, 11942 Jordan

**Keywords:** Body Mass Index, Epidemiology, Global Burden of Disease, Gout, Inflammatory arthritis, Mendelian randomization

## Abstract

**Background:**

Gout is one of the most common inflammatory arthritides and represents a growing health burden worldwide. This study compares the disease burden of gout and its attributable risk factors between China and European Union (EU) countries from 1990 to 2023 using data from the Global Burden of Disease Study 2023 (GBD 2023). In addition, the study evaluates potential causal relationships between key risk factors and gout and projects future trends in disease burden.

**Methods:**

Using GBD 2023 data, we analyzed the epidemiology of gout in China and EU countries. Analyses included descriptive statistics and age- and sex-specific comparisons. Joinpoint regression models were used to calculate annual percentage changes (APC) and average annual percentage changes (AAPC) to assess long-term trends. An autoregressive integrated moving average (ARIMA) model was applied to project gout burden trends in China and EU countries from 2024 to 2040. In addition, a two-sample Mendelian randomization (MR) approach was used to investigate the potential causal relationship between key risk factors and gout at the genetic level.

**Results:**

In 2023, China's age-standardized incidence rate (ASIR), prevalence rate (ASPR), and disability-adjusted life year rate (ASDR) for gout were 151.27/100,000, 809.69/100,000, and 25.14/100,000, respectively, all higher than in 1990. In comparison, EU countries showed lower levels for these indicators in both 1990 and 2023. Joinpoint regression analysis demonstrated an overall increasing trend in gout burden in both China and the EU between 1990 and 2023, although China experienced a brief decline in APC between 1990 and 1994. The burden of gout was higher among males than among females. Projections suggest that ASIR, ASPR, and ASDR will continue to increase in both China and European countries between 2024 and 2040. Mendelian randomization analysis further indicated a significant positive causal relationship between body mass index (BMI) and gout.

**Conclusion:**

This study combines GBD 2023 epidemiological data with Mendelian randomization analysis to characterize trends in the burden of gout in China and EU countries. The findings show a continuing increase in gout burden over time, particularly in China. The identified causal association between elevated BMI and gout highlights the importance of addressing modifiable metabolic risk factors to help reduce the future burden of gout.

**Table:** 

**Key Points** • *An increasing burden of gout could be observed in China and the European Union from 1990 to 2023.* • *A higher age-standardized burden of gout was observed in China than in the European Union.* • *Future projections indicate that the burden of gout will continue to increase through 2040.* • *A causal association between elevated body mass index and gout risk was supported by Mendelian randomization analysis.*

**Supplementary Information:**

The online version contains supplementary material available at 10.1007/s10067-026-08135-6.

## Introduction

Gout, an inflammatory arthritis caused by the deposition of monosodium urate crystals in joints and surrounding tissues, is a highly prevalent condition worldwide and represents a major public health concern that poses substantial challenges to population health. It has been designated as a priority chronic disease for prevention and control in many countries [1]. Recent epidemiological reports indicate that, globally, the prevalence of gout ranges from approximately 1% to 6.8%, while its annual incidence is estimated to be between 0.58 and 2.89 cases per 1,000 person-years [2]. Globally, both the prevalence and incidence of gout have shown a steady increase. Notably, this increase varies substantially across regions, with Asian countries demonstrating particularly high heterogeneity in gout prevalence. Recent epidemiological data from China further confirm that the prevalence of gout in the country is increasing markedly [3].

Because early gout often presents as intermittent acute arthritis or as asymptomatic hyperuricemia [4], many patients do not receive timely, guideline-recommended early interventions; as a result, recurrent flares and persistent hyperuricemia can progress over years into chronic tophaceous gout with tophi formation, irreversible joint destruction, and functional impairment [5]. Even when acute flares are controlled, a substantial proportion of patients experience recurrent attacks, joint deformities, and a high prevalence of cardiometabolic comorbidities, which together markedly increase the long-term disease burden [6–8]. Although advances in urate-lowering therapies and biologic agents have expanded therapeutic options, gaps remain in treatment adherence, individualized serum urate targets, and comprehensive management of comorbid conditions, which may limit improvements in population-level outcomes [9, 10].

Current gout-related burden analyses based on GBD data have primarily focused on global trends [11, 12] and country- or region-specific assessments, including studies from China [13], South Korea [14], North Africa and the Middle East [15], and various parts of Asia [16]. In addition, some comparative studies have examined differences between China and the global population [17]. However, research comparing gout trends between China and other individual countries remains limited, and cross-national analyses exploring regional heterogeneity are scarce. Moreover, most existing studies have primarily focused on descriptive assessments of disease burden, while evidence integrating population-level burden analyses with causal inference on risk factors remains limited. Although observational studies have suggested that high body mass index is associated with an increased risk of gout, these associations may be influenced by confounding and reverse causation. Mendelian randomization (MR), which uses genetic variants as instrumental variables, provides a useful approach for assessing potential causal relationships. To date, no comprehensive studies have systematically compared the gout burden between China and EU member states using the most recent GBD 2023 data.

The European Union (EU), comprising multiple high-income and upper-middle-income countries with substantial diversity in demographic structures, lifestyles, and healthcare systems, provides a relevant context for examining the burden and management of gout [18]. Given the marked heterogeneity between the EU and China in terms of socioeconomic development, healthcare systems, and the distribution of risk factors, comparing the gout burden between these regions can reveal important epidemiological differences and offer evidence to inform region-specific prevention and control strategies.

Therefore, using data from the most recent Global Burden of Disease Study 2023 (GBD 2023), this study aims to systematically compare the burden of gout and its attributable risk factors between China and EU member states from 1990 to 2023. We employed Joinpoint regression analysis to quantitatively assess temporal trends in gout-related indicators and to examine the evolution of disease burden across age and sex groups over the past three decades. Predictive models were constructed to project trends in incidence, prevalence, and disability-adjusted life years (DALYs) for the next 17 years in both China and the EU. In addition, MR analysis was employed to explore the potential causal relationship between high body mass index and gout, thereby providing complementary evidence to the population-level burden analysis.

## Methods

### Data source

This study represents a secondary analysis based on publicly available summary data from the GBD 2023. As a comprehensive global health assessment, GBD 2023 incorporates updated epidemiological information and standardized methodologies to systematically evaluate the health burden associated with 371 diseases and injuries and 88 attributable risk factors across 204 countries and territories from 1990 to 2023. Drawing on an extensive body of data sources, the study provides estimates of key indicators, including incidence, prevalence, mortality, years lived with disability (YLDs), years of life lost (YLLs), DALYs, and healthy life expectancy (HALE). In the GBD framework, gout is defined based on standardized case definitions mapped to ICD-10 code M10, which does not differentiate between crystal-proven gout and clinically diagnosed cases across regions. Therefore, potential differences in clinical diagnostic practices between China and EU countries may not be fully captured.

In this study, age-standardized incidence, prevalence, and DALY rates for gout in China and EU member states from 1990 to 2023 were retrieved and analyzed using the Global Health Data Exchange (GHDx) query tool (http://ghdx.healthdata.org/). Gout cases were identified using the International Classification of Diseases, 10th Revision (ICD-10) code M10. Because the data used in this study were obtained from publicly accessible databases, neither ethical approval nor informed consent was required.

In this study, the EU was analyzed as a single analytical unit to facilitate comparison with China at a broader regional level, consistent with the regional aggregation approach used in the GBD framework.

### Statistical analysis

Using data from the GBD 2023, this study extracted incidence, prevalence, and DALYs related to gout for China and EU member states, along with their corresponding age-standardized indicators—age-standardized incidence rate (ASIR), age-standardized prevalence rate (ASPR), and age-standardized DALY rate (ASDR). All estimates are reported with their respective 95% uncertainty intervals (UIs).

To assess long-term temporal patterns, Joinpoint regression analysis was used. By fitting segmented regression lines, this model partitions the study period into intervals with distinct trend characteristics and objectively identifies statistically significant trend inflection points. We calculated the annual percent change (APC) for each segment and the average annual percent change (AAPC) for the entire study period, along with their corresponding 95% confidence intervals (CIs).

The criteria for determining trend direction were as follows: if the 95% CI of the AAPC was entirely above zero, the trend was considered increasing; if it was entirely below zero, the trend was considered decreasing; and if the 95% CI included zero, the trend was regarded as stable.

This approach enables a more accurate depiction of the nonlinear evolution of gout incidence trends in China and EU countries, minimizing potential biases associated with traditional linear trend analyses [19, 20].

We used an autoregressive integrated moving average (ARIMA) model to forecast the incidence, prevalence, and DALY rates of gout in China and EU member states from 2024 to 2040. As a classical time-series analytical approach, the ARIMA model is widely used in epidemiological forecasting studies and is well suited for evaluating both stationary and non-stationary time-series data [21–23]. It enables the identification of underlying temporal patterns based on historical observations and has been frequently applied to project disease burden trends when long-term continuous surveillance data are available [24].

In the ARIMA (p, d, q) model, the parameter p denotes the order of the autoregressive component, d represents the degree of differencing required to achieve stationarity, and q indicates the order of the moving-average component. Model development—including series stabilization, model identification, parameter estimation, and diagnostic checking—was conducted following standard procedures commonly reported in prior time-series forecasting studies, and model adequacy was assessed through residual diagnostics [25].

All statistical analyses and data visualizations in this study were performed using R software (version 4.2.3). Joinpoint regression was conducted using the Joinpoint Regression Program (version 5.2.0).

### Risk factors

Relevant risk factors were obtained through the GHDx platform. Within the assessment framework of the GBD 2023, the burden of gout attributable to major risk factors—particularly high BMI and kidney dysfunction—was systematically quantified for the period from 1990 to 2023 [26].

Detailed definitions, exposure estimations, and attributable burden calculations for high BMI and kidney dysfunction within the GBD framework are available in previously published methodological reports of the GBD study [27].

Building on these established methods, the present analysis quantified the percentage contribution of these two risk factors to the total number of gout-related DALYs in China and EU member states in 1990 and 2023.

### Mendelian randomization analysis

According to the GBD 2023 report, high BMI is a risk factor for gout in addition to renal impairment. Therefore, this study conducted a confirmatory analysis of the causal relationship between BMI and gout.

Five MR approaches were implemented in this study. The inverse variance weighting (IVW) method was used as the primary analytical strategy, while the other four methods served as complementary analyses [28]. A P value < 0.05 was considered indicative of a causal association between the exposure and the outcome, and an OR > 1 was interpreted as evidence of a positive relationship. The IVW estimates were regarded as the main results of the MR analysis.

In addition, heterogeneity and pleiotropy tests were performed to assess the robustness and reliability of the findings.

### Instrumental variables and data screening

The instrumental variables (IV) used in this MR analysis were derived from publicly available genome-wide association studies (GWAS) databases.

Specifically:

- IV for BMI (exposure): ieu-b-40.
- IV for gout (outcome): ieu-a-1054.

Detailed information on all instrumental variables is provided in Supplementary Table S3. MR analysis uses single nucleotide polymorphisms (SNPs) as IVs to infer causal relationships between exposure and outcome. Its validity relies on three core assumptions: the correlation assumption, the independence assumption, and the exclusion restriction assumption.

To construct valid instrumental variables, we applied the following screening criteria:

- Significance threshold: P < 5 × 10⁻⁸ was applied to all datasets.
- Linkage disequilibrium (LD) clustering: Parameters r^2^ < 0.001 and distance > 10,000 kb were set to exclude highly linked SNPs.
- Instrument strength: The F-statistic was calculated, retaining only SNPs with F > 10 to avoid weak instrument bias.

Additionally, to assess robustness and control for potential bias, we performed leave-one-out analysis. The MR-Steiger test was applied to validate the direction of causality estimates for each SNP. The Phenoscanner test was used to screen each SNP for potential associations with confounding factors that might violate the independence assumption, and such SNPs were excluded.

## Results

### Overall temporal trends in gout incidence, prevalence, and DALYs across China and the EU

Overall, the number of new gout cases in China increased markedly from 1,188,962 (95% UI: 949,192–1,476,316) in 1990 to 3,215,472 (95% UI: 2,529,611–4,037,875) in 2023, representing a 170.44% increase. During the same period, the number of prevalent cases rose by 194.11%, reaching 17,672,308 individuals in 2023. The total DALYs attributable to gout increased from 188,934 in 1990 to 545,814 in 2023, an overall rise of 188.89%. Consistently, the age-standardized indicators—ASIR, ASPR, and ASDR—showed sustained upward trends. From 1990 to 2023, the AAPC for ASIR was 0.98 (95% CI: 0.87–1.08), for ASPR was 1.08 (95% CI: 0.97–1.19), and for ASDR was 1.07 (95% CI: 0.96–1.17). In contrast, although the EU also experienced increases in ASIR, ASPR, and ASDR between 1990 and 2023, the overall levels of these indicators remained substantially lower than those observed in China during the same period (Table 1).

**Table 1:** All-ages cases, age-standardized incidence, prevalence, DALYs rate, and corresponding AAPC of gout in China and EU countries in 1990 and 2023

		1990		2023		1990–2023 AAPC
		All-ages cases	Age-standardized rates per 100 000 people	All-ages cases	Age-standardized rates per 100 000 people	
Location	Measure	*n* (95% UI)	*n* (95% UI)	*n* (95% UI)	*n* (95% UI)	*n* (95% CI)
China	Incidence	1,188,962 (949,192–1,476,316)	122.47 (98.09–153.00)	3,215,472 (2,529,611–4,037,875)	151.27 (120.46–189.12)	0.98 (0.87–1.08)
	Prevalence	6,008,711 (4,795,809–7,613,438)	640.47 (511.00–801.13)	17,672,308 (13,878,800–22,353,430)	809.69 (647.27–1009.55)	1.08 (0.97–1.19)
	DALYs	188,934 (125,368–268,975)	19.88 (13.26–28.28)	545,814 (363,327–779,105)	25.14 (16.72–35.52)	1.07 (0.96–1.17)
EU	Incidence	406,200 (321,579–505,606)	73.80 (59.22–92.09)	640,251 (499,057–803,136)	82.77 (66.03–103.37)	0.30 (0.27–0.32)
	Prevalence	2,780,639 (2,202,534–3,511,882)	487.99 (390.48–611.50)	4,787,329 (3,753,531–6,018,034)	568.52 (454.55–702.63)	0.48 (0.45–0.51)
	DALYs	85,416 (57,473–121,565)	15.09 (10.21–21.37)	145,184 (97,609–208,761)	17.56 (11.84–25.04)	0.48 (0.45–0.51)

*DALYs* disability-adjusted life years, AAPC average annual percentage change, *EU* European Union, *UI* uncertainty interval, *CI* confidence interval

### Joinpoint regression analysis of the gout burden in China and the EU

Joinpoint regression analysis revealed distinct temporal patterns in the gout burden for China and EU member states.

**Fig. 1:**
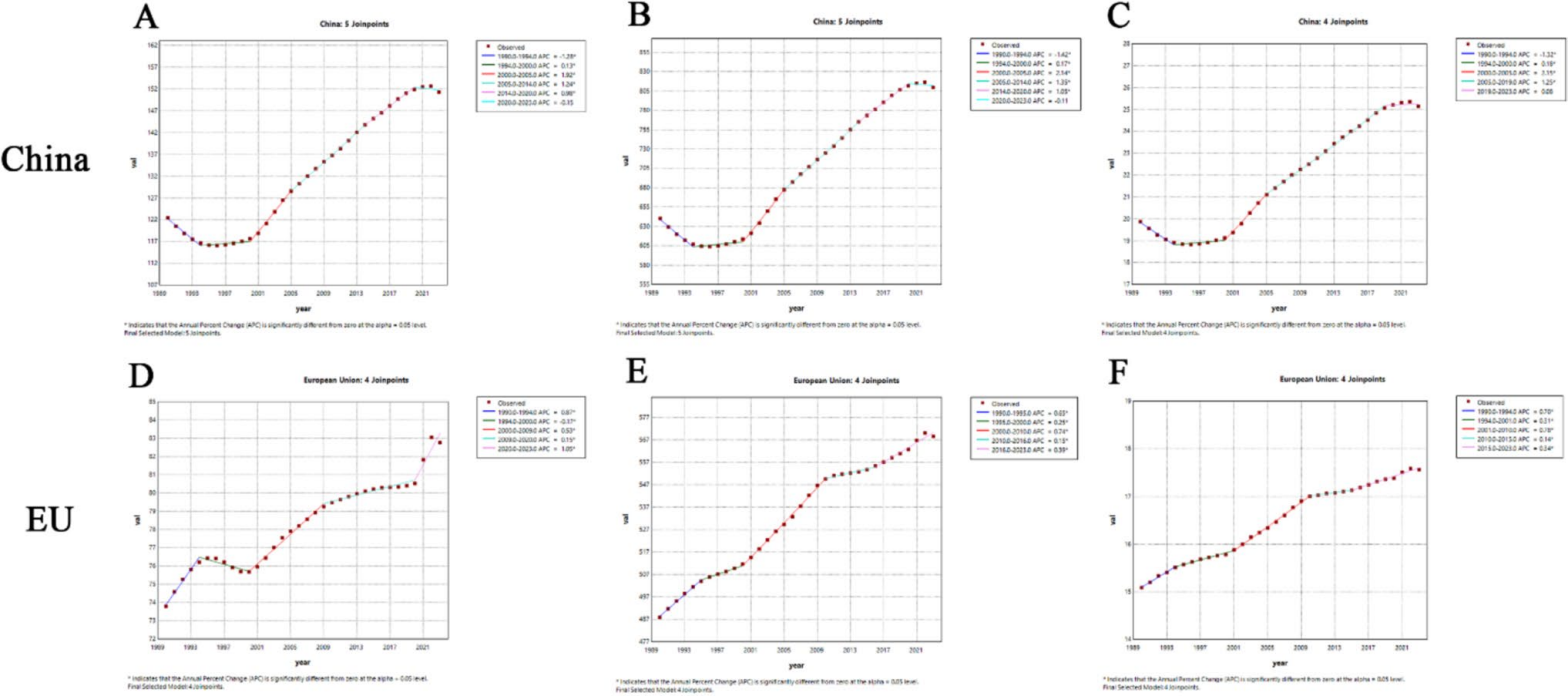
Trends in ASIR, ASPR, and ASDR of gout in China and the EU between 1990 and 2023, assessed using joinpoint regression models (*P < 0.05). (**A**) Joinpoint regression results for gout ASIR in China. (**B**) Joinpoint regression results for gout ASPR in China. (**C**) Joinpoint regression results for gout ASDR in China. (**D**) Joinpoint regression results for gout ASIR in the EU. (**E**) Joinpoint regression results for gout ASPR in the EU. (**F**) Joinpoint regression results for gout ASDR in the EU

China: From 1990 to 1994, the ASIR, ASPR, and ASDR all showed significant declining trends (ASIR: APC =  − 1.28, *P* < 0.05; ASPR: APC =  − 1.42, *P* < 0.05; ASDR: APC =  − 1.32, *P* < 0.05). During the period from 1994 to 2000, these indicators entered a relatively stable phase, with no statistically significant changes observed. From 2000 to 2023, all three indicators shifted to significant upward trends (*P* < 0.05) **(**Fig. 1** A-C)**.

European Union: From 1990 to 1994, ASIR exhibited a significant increasing trend (APC = 0.87, *P* < 0.05). This was followed by a significant decline between 1994 and 2000 (APC =  − 0.17, *P* < 0.05). From 2000 to 2023, ASIR again showed a significant upward trend (*P* < 0.05). Over the entire study period from 1990 to 2023, both ASPR and ASDR in the EU demonstrated persistent and significant increasing trends (*P* < 0.05) (Fig. 1 D-F).

### Temporal trends in the burden of gout in China and the EU

**Fig. 2:**
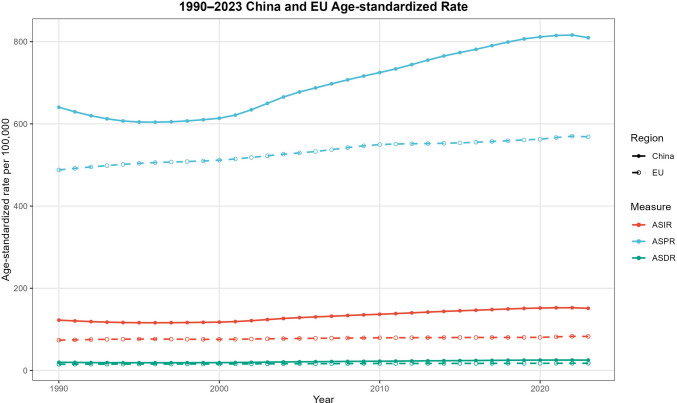
Comparative analysis of temporal trends in ASIR, ASPR and ASDR of gout in China and the EU between 1990 and 2023

From 1990 to 2023, the overall ASPR of gout increased in both China and the EU. Specifically, in China, ASPR showed a slight decline between 1990 and 1996, followed by a gradual upward trend thereafter. In contrast, ASPR in the EU increased steadily throughout the study period. In addition, both the ASIR and ASDR exhibited modest upward trends in China and the EU from 1990 to 2023 **(**Fig. 2**)**.

### Age-specific and sex-specific differences in gout burden in China and the EU

**Fig. 3:**
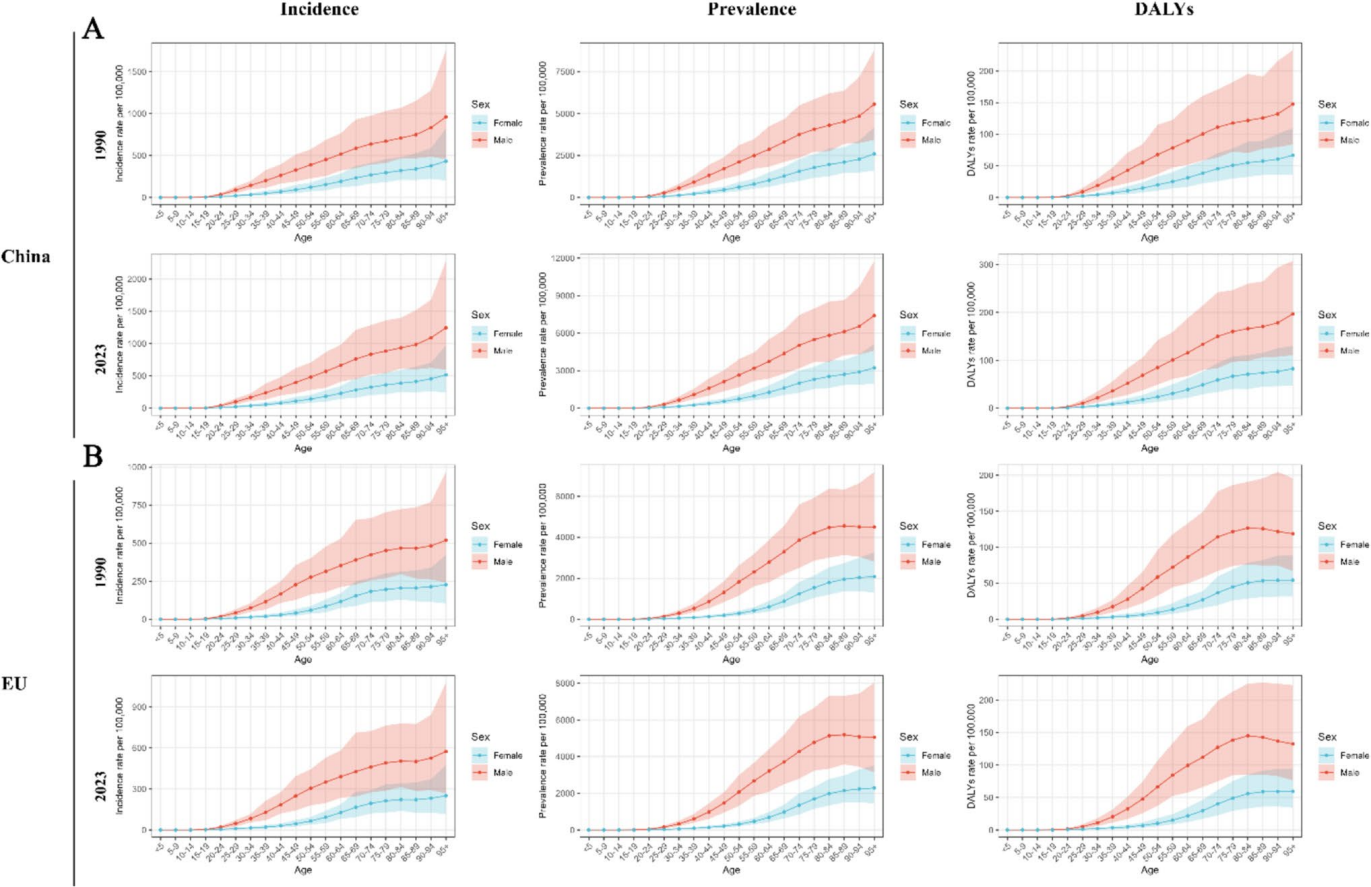
Comparison of ASIR, ASPR, and ASDR across different age groups in China and the EU in 1990 and 2023. (**A**) ASIR, ASPR, and ASDR by age group in China for 1990 and 2023. (**B**) ASIR, ASPR, and ASDR by age group in the EU for 1990 and 2023

**Fig. 4:**
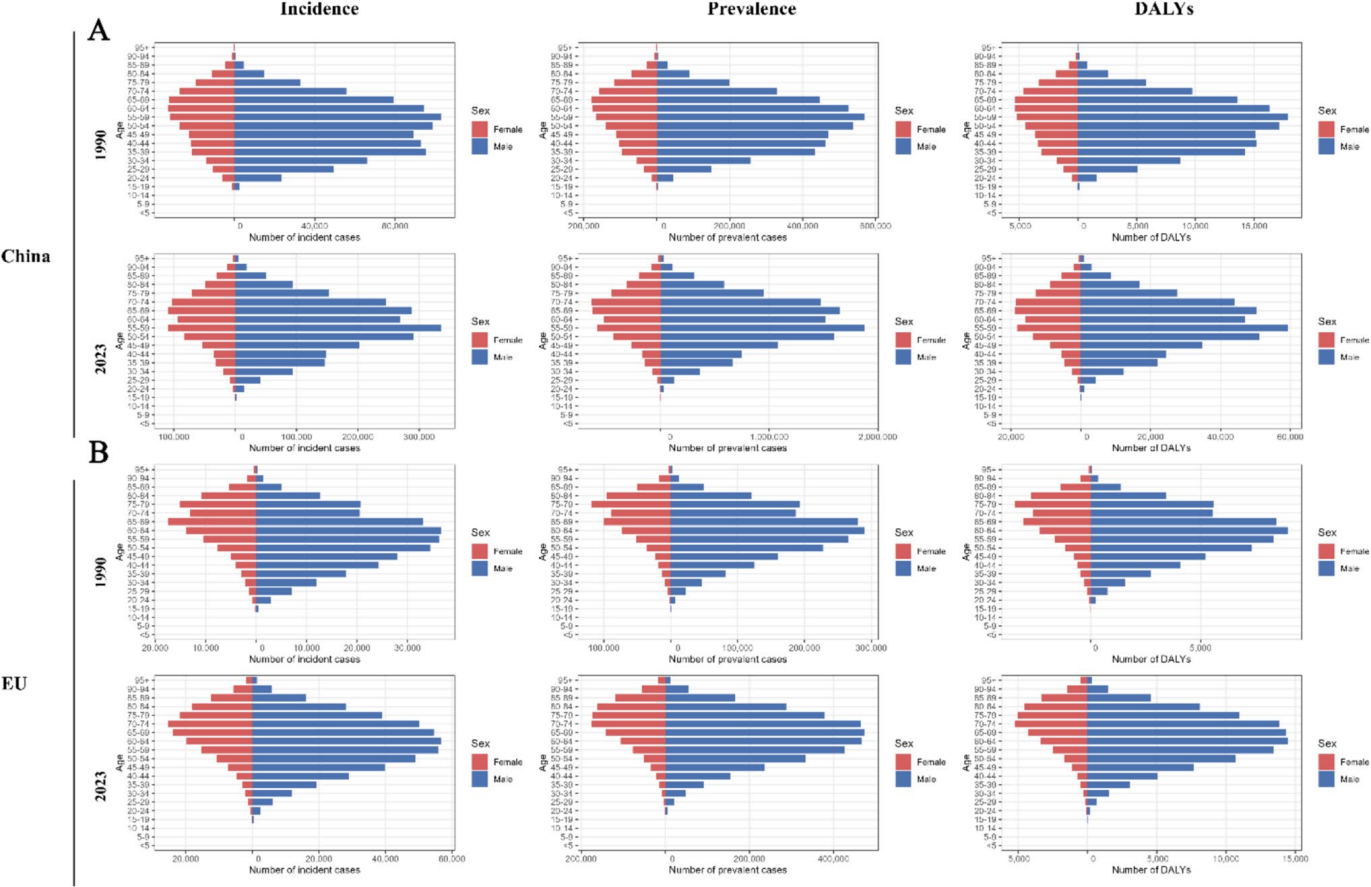
Numbers of incident cases, prevalent cases, and DALYs for different age groups with gout in China and the EU in 1990 and 2023. (**A**) Incident cases, prevalent cases, and DALYs by age group in China for 1990 and 2023. (**B**) Incident cases, prevalent cases, and DALYs by age group in the EU for 1990 and 2023

Figures 3 and 4 depict the distributions of the gout burden across age and sex groups in China and EU member states in 1990 and 2023, presenting age-standardized rates **(**Fig. 3**)** and absolute numbers **(**Fig. 4**)**, respectively. Specifically, Fig. 3 illustrates the ASIR, ASPR, and ASDR stratified by age group and sex, whereas Fig. 4 shows the corresponding estimated numbers of incident cases, prevalent cases, and DALYs in these populations.

In both 1990 and 2023, ASIR, ASPR, and ASDR of gout in China and the EU showed pronounced age- and sex-specific differences. Overall, all three indicators began to increase after the age of 25 years in both sexes, with consistently higher levels observed in males than in females. In China, the age-specific patterns of ASIR, ASPR, and ASDR were similar in 1990 and 2023. All three indicators increased steadily with advancing age, and the highest values for both males and females were observed in the ≥ 95-year age group. In the EU, the age distribution of ASIR closely resembled that observed in China. By contrast, different age-specific peak patterns were observed for ASPR and ASDR in the EU. For ASPR, prevalence among males peaked in the 85–89-year age group in both 1990 and 2023 and then declined slightly, whereas ASPR among females continued to increase with age. For ASDR, rates among males reached a peak in the 80–84-year age group and subsequently declined, while ASDR among females increased with age and peaked in the 90–94-year age group before remaining relatively stable.

**Fig. 5:**
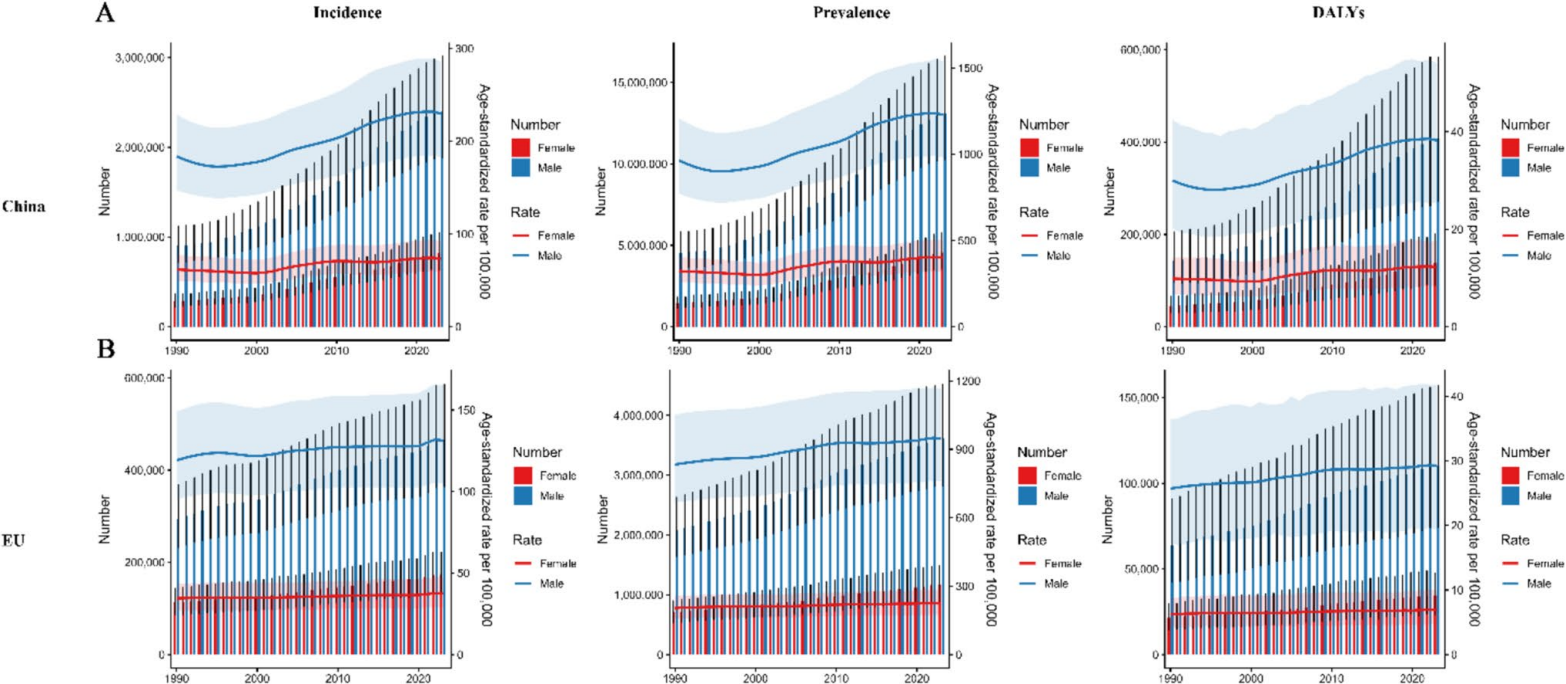
Case numbers and age-standardized rates (ASIR, ASPR, and ASDR) for males and females of all ages in China and the EU from 1990 to 2023. (**A**) Case numbers and ASIR, ASPR, and ASDR for males and females of all ages in China. (**B**) Case numbers and ASIR, ASPR, and ASDR for males and females of all ages in the EU. Bars indicate counts, and lines indicate age-standardized rates

Figure 5 presents the incidence, prevalence, and DALYs of gout in China and EU countries from 1990 to 2023 for all ages, along with their corresponding age-standardized rates (ASIR, ASPR, ASDR). Overall, both the ASIR and the number of gout cases in China showed a significant upward trend, with males consistently exhibiting higher case numbers and ASIR than females. The trends in prevalence and DALYs mirrored these patterns. In contrast, the EU showed a much slower increase in both case numbers and age-standardized rates, a trend that was particularly evident among females.

### Forecasts of gout incidence, prevalence, and DALYs in China and the EU, 2024–2040

**Fig. 6:**
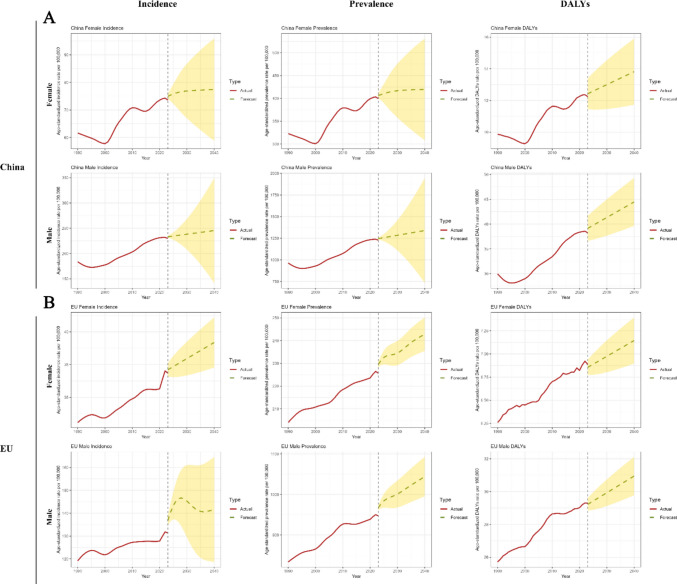
Trends in the ASIR, ASPR, and ASDR of gout in China and the EU from 1990 to 2023, with estimated projections from 2024 to 2040. (**A**) Changes in ASIR, ASPR, and ASDR of gout in China during 1990–2023 and projected trends for 2024–2040. (**B**) Changes in ASIR, ASPR, and ASDR of gout in the EU during 1990–2023, with estimated projections from 2024 to 2040

We applied ARIMA models to sex-stratified ASIR, ASPR, and ASDR of gout from 1990 to 2023. Based on these models, trends from 2024 to 2040 were projected. Model parameters were selected using the Akaike Information Criterion (AIC) and the Bayesian Information Criterion (BIC) (Table S2). The projections indicate that ASIR, ASPR, and ASDR will continue to increase in both China and the EU through 2040 (Fig. 6, Table S1).

China: Among males, ASIR is projected to increase from 233.90 per 100,000 to 245.48 per 100,000 (5.00%). ASPR is expected to rise from 1251.27 to 1339.03 per 100,000 (7.01%). ASDR is projected to increase from 39.45 to 44.47 per 100,000 (12.72%). Among females in China, ASIR is expected to increase from 75.39 to 77.37 per 100,000 (2.63%). ASPR is projected to rise from 408.34 to 419.72 per 100,000 (2.79%). ASDR is expected to increase from 12.51 to 13.82 per 100,000 (10.42%).

EU: Among males, ASIR is projected to increase from 140.36 to 141.64 per 100,000 (0.91%). ASPR is expected to rise from 977.09 to 1044.14 per 100,000 (6.89%). ASDR is projected to increase from 29.32 to 30.95 per 100,000 (5.56%). Among females in the EU, ASIR is expected to increase from 37.78 to 39.35 per 100,000 (4.16%). ASPR is projected to rise from 231.37 to 242.85 per 100,000 (4.96%). ASDR is expected to increase from 6.87 to 7.14 per 100,000 (3.93%).

### Distribution of risk factor–attributable gout DALYs in China, the EU and EU countries

**Fig. 7:**
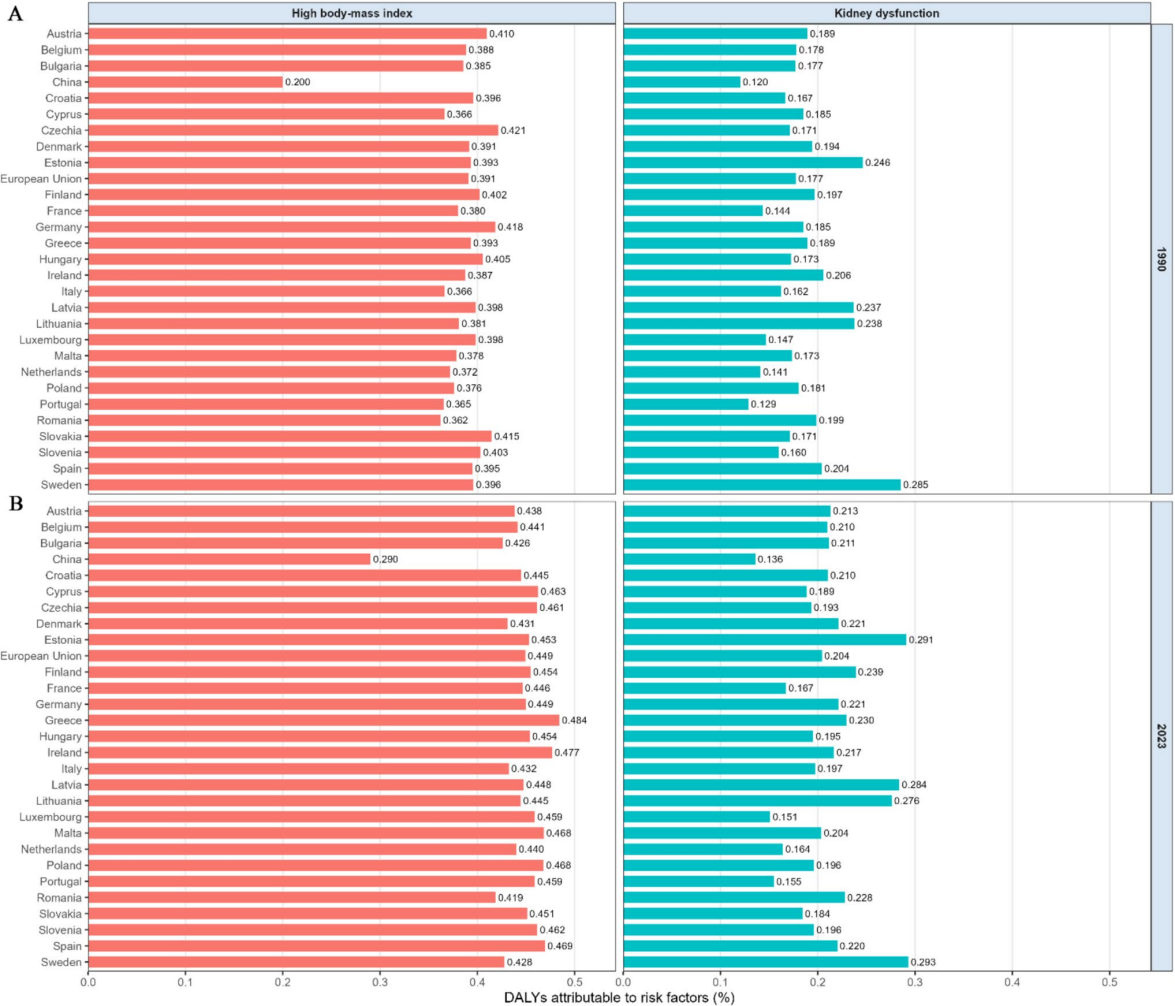
Distribution of attributable risk factors for gout-related DALYs in China, the EU, and EU countries in 1990 and 2023. (**A**) Proportional contributions of attributable risk factors to gout DALYs in China, the EU, and EU countries in 1990. (**B**) Proportional contributions of attributable risk factors to gout DALYs in China, EU and EU countries in 2023

Figure 7 illustrates the proportions of gout-related disease burden attributable to different risk factors in China, the EU, and EU member states in 1990 and 2023. The results show that high body mass index and kidney dysfunction were the leading risk factors across all regions. However, their relative contributions varied substantially between countries, with particularly marked differences observed for kidney dysfunction. In 1990, the three countries with the highest proportions of gout DALYs attributable to kidney dysfunction were Sweden (0.285%), Estonia (0.246%) and Lithuania (0.238%). China had the lowest proportion (0.120%). By 2023, Sweden (0.293%), Estonia (0.291%), and Latvia (0.284%) ranked highest. Notably, compared with 1990, all countries showed an increase in the proportion of DALYs attributable to kidney dysfunction in 2023.

Notably, cross-country differences in the proportion of gout burden attributable to high body mass index were relatively small. Compared with 1990, Greece had the highest proportion of gout-related DALYs attributable to high body mass index in 2023 (0.484%) among the countries examined. By 2023, this proportion had increased in all countries compared with 1990.

### MR analysis results

**Fig. 8:**
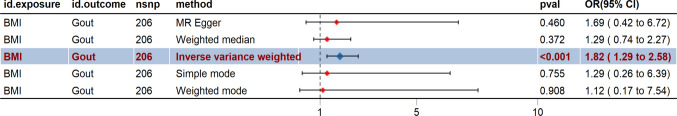
Forest plot of Mendelian randomization analysis showing the association between body mass index and gout

This study conducted MR analysis to assess the relationship between BMI and gout. Table S3 presents detailed information on the instrumental variables for the exposure and outcome. Results from the IVW analysis indicated a positive causal association between BMI (OR = 1.82, 95% CI: 1.29–2.58, P < 0.001) and gout (Fig. 8 and Table S4). A slope greater than zero indicates that BMI is a detrimental factor for gout, and this is consistent across all five methods employed (Fig. S1). As shown in Fig. S2, the IVW estimate is positioned to the right of the null line, further supporting a positive causal association.

For the sensitivity analysis of BMI, Fig. S3 shows that after excluding each SNP, the overall confidence intervals remained entirely to the right of zero, indicating the robustness of the results. Fig. S4 and Table S5 show the heterogeneity analysis of BMI results. The points on either side of the IVW line are approximately symmetrical, indicating the absence of heterogeneity in the results. To verify the causal direction between exposure and outcome, we conducted MR-Steiger tests. As shown in Table S6, all instrumental variable tests yielded P < 0.05, indicating that the causal direction from BMI as an exposure factor to gout outcomes is correct and robust, thereby ruling out the possibility of reverse causality.

Furthermore, the MR-Egger intercept test was performed to evaluate potential directional horizontal pleiotropy. The intercept was close to zero (intercept = 0.0011, SE = 0.0098) and not statistically significant (P = 0.91), suggesting no evidence of directional horizontal pleiotropy (Table S7). These findings support the robustness and reliability of the MR results.

## Discussion

Gout, an inflammatory disease that seriously threatens human health worldwide, has become a major public health challenge [29]. The GBD study provides valuable data and methodological frameworks for systematically investigating disease burden, predicting its evolution, and conducting cross-national comparisons [30]. An in-depth analysis of long-term epidemiological trends of gout in China, together with comparisons with other countries in a global context, is of considerable practical importance for informing public health strategies suited to China's national conditions, strengthening international cooperation in disease prevention and control, and ultimately improving population health.

However, existing studies rarely systematically analyze the disease burden of gout in China within an international comparative context, and its latest epidemiological characteristics and global positioning remain unclear. Therefore, this study, based on GBD 2023 data, systematically compares temporal trends of gout incidence, prevalence, and DALYs across different age and sex dimensions in China and the EU from 1990 to 2023. It also analyzes the main attributable risk factors for DALYs and predicts the disease burden from 2024 to 2040. To our knowledge, this study provides a systematic comparison of the disease burden of gout between China and the European Union countries based on the latest GBD 2023 data. In addition, by combining population-level disease burden analysis with causal inference from Mendelian randomization, this study provides complementary evidence regarding the relationship between high body mass index and gout in Chinese and European populations.

Consistent with previous studies, our findings indicate a sustained increase in the burden of gout in both China and the EU over the past decades(31). Based on the ARIMA forecasting model, gout incidence, prevalence, and DALY rates in China are projected to continue increasing in the coming decades, based on historical trend estimates.

In recent years, the disease burden of gout has been on the rise in both China and the EU. However, compared with the EU, the increase in the incidence, prevalence, and DALYs associated with gout in China is particularly pronounced. Notably, our study found that, both in 1990 and 2023, the ASIR, ASPR, and ASDR of gout in China were higher than those in the EU during the same periods. These differences may be explained by variations in healthcare systems, lifestyle patterns, and the management of chronic diseases across regions. EU countries generally have more established chronic disease management systems, which may contribute to better control of gout-related risk factors(32–36).

High body mass index is a known risk factor for gout [37]. Current research suggests that a high body mass index may contribute to hyperuricemia, chronic inflammation, and insulin resistance, which are associated with gout development [38, 39].

Furthermore, despite a higher absolute burden of gout in China, the GBD-estimated population attributable fraction of gout due to kidney dysfunction remained consistently lower than that observed in EU countries in both 1990 and 2023. This finding suggests that gout in China is less predominantly driven by chronic kidney impairment and more likely influenced by other risk factors such as dietary patterns, alcohol consumption, and genetic susceptibility. In addition, genetic variants affecting urate transport and excretion (such as polymorphisms in the ABCG2 and SLC2A9 genes), which have been reported to occur more frequently in East Asian populations, may also contribute to the higher susceptibility to gout observed in China [40, 41]. In contrast, the higher attribution to kidney dysfunction in EU countries may reflect a higher prevalence of chronic kidney disease, and a greater burden of metabolic comorbidities, indicating a more pronounced role of renal impairment in gout pathogenesis.

The results of the Joinpoint regression model indicate that between 1990 and 2023, the ASIR, ASPR, and ASDR of gout in China generally showed an upward trend, but a downward trend between 1990 and 1994. The reason for these decreases is unclear, but it may be related to the systemic changes in China's disease detection and registration system, namely the transition from an older, potentially inconsistent, disease classification method to the internationally standardized ICD-10 coding system. During this transition period (approximately 1990–1994), unfamiliarity with coding, system instability, and adjustments to the reporting network led to an artificially low level of gout statistics, creating the illusion of a "decline" [42].

The burden of gout also exhibits sex and age differences. The higher burden of gout among males observed in both China and the EU is consistent with previous studies and may be attributed to hormonal, behavioral, and lifestyle differences, including dietary habits and alcohol consumption, as well as the protective effects of estrogen in females [43–48].

Our findings indicate that developing precise prevention and treatment strategies based on gender differences is crucial for reducing the incidence of gout. Meanwhile, with the accelerating aging of China's population, this study found that the incidence, prevalence, and DALY rate of gout all increase significantly with age. Since the elderly often suffer from multiple systemic diseases, their gout-related health complications are also more complex. Given the continued increase in the proportion of elderly people in China, it is expected that there will be more gout cases and a heavier disease burden in the future. Therefore, strengthening health education and comprehensive management for the elderly population is important for the prevention and control of gout.

Importantly, building on the GBD findings that identified high BMI as a major contributor to gout-related DALYs, the MR analysis provides genetic evidence supporting a causal relationship between BMI and gout. This genetic evidence strengthens the interpretation of the epidemiological trends observed in the GBD analysis. Compared with prior MR studies, our analysis reinforces the causal role of BMI in gout, while integrating population-level burden estimates, thus providing complementary insights into epidemiological trends.

Together, these results indicate that the rising prevalence of obesity may be a driver of the increasing burden of gout in both China and the EU. Therefore, integrating obesity prevention and weight management into public health strategies may be important for reducing the burden of gout. These findings are broadly consistent with previous global analyses showing that high BMI contributes substantially to gout burden, such as studies in South Korea and North America [49, 50]. However, our study provides updated estimates based on GBD 2023 and adds a cross-national comparison between China and EU, highlighting regional heterogeneity.

From an epidemiological perspective, the rising prevalence of obesity appears to parallel the increasing burden of gout observed in both China and the EU (51, 52). Given that obesity contributes to hyperuricemia through mechanisms such as insulin resistance and altered renal urate handling, the temporal trends of these two conditions may be closely linked [53, 54]. This suggests that the global increase in obesity may be an important driver of the rising incidence and prevalence of gout.

Comparing China with EU member states highlights opportunities to adopt best practices from regions with more effective chronic disease management, which can guide national policy and resource allocation. Given the projected increase in gout burden, the demand for urate-lowering therapies is also expected to rise substantially. Considering that a proportion of patients may not tolerate first-line treatments, healthcare systems may need to prepare for increased use of alternative therapies to ensure adequate disease control in the majority of patients. This highlights the importance of optimizing treatment strategies and improving access to a broader range of therapeutic options.

### Limitations

This study has several limitations. First, although the data used in this study were obtained from the GBD 2023 database—an authoritative source of global disease burden estimates—data scarcity and the reliance on model-based estimations remain inherent limitations of the GBD framework and may not fully reflect the true epidemiological characteristics of gout. In addition, the projections generated by the ARIMA model are based on historical trends and do not explicitly account for potential future changes in demographic structure, healthcare policies, or risk factor distributions. Therefore, the forecasting results should be interpreted as trend-based projections rather than precise predictions.

Second, due to the lack of detailed data on gout subtypes (such as primary and secondary gout) and disease severity or clinical complications in the GBD 2023 database, we were unable to comprehensively assess the disease burden of gout from a clinical or pathological perspective. In addition, variations in diagnostic practices between regions, such as differences in the use of crystal confirmation versus clinical diagnosis, may introduce heterogeneity that is not fully accounted for in the GBD estimates. Furthermore, gout may be underdiagnosed in some populations, particularly in regions with limited healthcare access or lower disease awareness, which may lead to an underestimation of the true disease burden. Existing studies have shown significant differences in prognosis and treatment needs among different types or severities of gout [55, 56].

Third, this study focused primarily on high body mass index and renal dysfunction as the main attributable factors for gout, while the GBD 2023 database does not cover other potential risk factors (such as dietary structure, alcohol intake, etc.), therefore we were unable to assess the contribution of these factors to the disease burden. In addition, the EU was analyzed as a single analytical unit in this study. Given the heterogeneity among EU member states in terms of healthcare systems, dietary patterns, and demographic structures, the aggregated results may mask country-level variations.

Fourth, due to differences in GBD data sources and estimation methods, our results may differ somewhat from other studies, but the overall trend is generally consistent with existing literature. The GWAS data used in MR analyses primarily originate from European populations, which may not fully represent the populations included in the GBD analysis, particularly those from China. Therefore, the generalizability of the MR findings to other populations should be interpreted with caution. Gout is also influenced by multiple factors including genetic background, occupational exposures, and environmental factors, which were not incorporated in this study and warrant further investigation in future research. In addition, the lack of region-specific BMI data for China and the EU may limit the interpretability of the MR findings in a comparative context.

Finally, it should be noted that the population coverage and data quality of the GBD database have evolved over time. Therefore, our conclusions should be interpreted cautiously and considered alongside evidence from other epidemiological data sources.

## Conclusion

This study systematically analyzed and projected the disease burden and long-term trends of gout in China and the European Union. The results indicate that over the past three decades, the incidence, prevalence, and disability-adjusted life years (DALYs) associated with gout have increased in both regions. Compared with the EU, China currently shows a relatively higher burden of gout.

These findings suggest that future prevention and control strategies for gout in China may benefit from more targeted approaches. In particular, efforts should focus on controlling key modifiable risk factors such as elevated body mass index, as well as implementing tailored interventions based on sex- and age-specific risk patterns. In addition, improving public awareness of gout through health education, optimizing the quality of medical services, and promoting research on improved diagnostic and therapeutic approaches may help reduce the future incidence and overall burden of the disease. These findings highlight the need for coordinated public health strategies aimed at obesity control, early diagnosis, and improved management of gout in aging populations.

## Supplementary Information

Below is the link to the electronic supplementary material.

Supplementary file1 (DOCX 27 KB)

Supplementary file2 (DOCX 20 KB)

Supplementary file3 (DOCX 17 KB)

Supplementary file4 (XLSX 30 KB)

Supplementary file5 (DOCX 17 KB)

Supplementary file6 (DOCX 16 KB)

Supplementary file7 (DOCX 16 KB)

## Data Availability

The data utilized in this study are publicly accessible on the Institute for Health Metrics and Evaluation (IHME) website (https://ghdx.healthdata.org/gbd-2023).
