# Supplementary material for: Epidemiological trends and burden of gout in China and the European Union: a GBD 2023 and Mendelian randomization study
Source: Clin Rheumatol. 2026 May 5;45(6):3031–45. doi: 10.1007/s10067-026-08135-6 (PMC13249755; doi:10.1007/s10067-026-08135-6)
Supplement: Supplementary file 1 — Supplementary file1 (DOCX 27 KB) [file 10067_2026_8135_MOESM1_ESM.docx]

| **Table S1**  **Future projections of incidence rates, prevalence rates, and DALYs associated with gout in China and EU between 2024 and 2040** | | | | | | | | | |
| --- | --- | --- | --- | --- | --- | --- | --- | --- | --- |
|  |  | **Age-standardized incidence rate, per 100 000 people (95% CI)** | |  | **Age-standardized prevalence rate, per 100 000 people (95% CI)** | |  | **Age-standardized DALYs rate, per 100 000 (95% CI)** | |
| **Location** | **Year** | **Male** | **Female** |  | **Male** | **Female** |  | **Male** | **Female** |
| China | 2024 | 233.90 (229.64–238.17) | 75.39 (73.24–77.55) |  | 1251.27 (1227.43–1275.11) | 408.34 (395.60–421.07) |  | 39.45 (36.73–42.17) | 12.51 (11.43–13.58) |
|  | 2025 | 234.62 (227.39–241.85) | 75.80 (72.29–79.30) |  | 1256.59 (1215.88–1297.31) | 410.39 (389.63–431.14) |  | 39.77 (36.87–42.66) | 12.59 (11.42–13.76) |
|  | 2026 | 235.34 (224.51–246.17) | 76.14 (71.20–81.08) |  | 1261.99 (1200.64–1323.34) | 412.28 (382.93–441.63) |  | 40.08 (37.02–43.14) | 12.67 (11.42–13.92) |
|  | 2027 | 236.06 (221.07–251.06) | 76.41 (70.07–82.76) |  | 1267.44 (1182.09–1352.79) | 413.89 (376.12–451.67) |  | 40.39 (37.17–43.61) | 12.75 (11.43–14.08) |
|  | 2028 | 236.79 (217.12–256.45) | 76.62 (68.96–84.27) |  | 1272.91 (1160.53–1385.30) | 415.17 (369.56–460.78) |  | 40.71 (37.33–44.08) | 12.84 (11.43–14.24) |
|  | 2029 | 237.51 (212.73–262.29) | 76.76 (67.93–85.60) |  | 1278.40 (1136.23–1420.58) | 416.12 (363.39–468.85) |  | 41.02 (37.50–44.53) | 12.92 (11.45–14.39) |
|  | 2030 | 238.24 (207.91–268.56) | 76.87 (66.96–86.78) |  | 1283.90 (1109.41–1458.40) | 416.81 (357.60–476.02) |  | 41.33 (37.68–44.99) | 13.00 (11.46–14.54) |
|  | 2031 | 238.96 (202.71–275.21) | 76.96 (66.05–87.87) |  | 1289.41 (1080.25–1498.57) | 417.32 (352.11–482.54) |  | 41.65 (37.86–45.43) | 13.08 (11.48–14.68) |
|  | 2032 | 239.68 (197.15–282.22) | 77.03 (65.18–88.88) |  | 1294.92 (1048.91–1540.93) | 417.74 (346.83–488.65) |  | 41.96 (38.04–45.87) | 13.16 (11.50–14.82) |
|  | 2033 | 240.41 (191.24–289.57) | 77.10 (64.34–89.86) |  | 1300.43 (1015.51–1585.35) | 418.11 (341.68–494.54) |  | 42.27 (38.23–46.31) | 13.24 (11.52–14.97) |
|  | 2034 | 241.13 (185.02–297.25) | 77.17 (63.51–90.82) |  | 1305.94 (980.17–1631.72) | 418.45 (336.62–500.28) |  | 42.59 (38.42–46.75) | 13.33 (11.55–15.11) |
|  | 2035 | 241.86 (178.48–305.24) | 77.22 (62.69–91.75) |  | 1311.46 (942.97–1679.95) | 418.76 (331.64–505.89) |  | 42.90 (38.62–47.18) | 13.41 (11.57–15.24) |
|  | 2036 | 242.58 (171.65–313.52) | 77.27 (61.89–92.65) |  | 1316.97 (904.00–1729.94) | 419.04 (326.74–511.34) |  | 43.21 (38.82–47.61) | 13.49 (11.60–15.38) |
|  | 2037 | 243.31 (164.54–322.08) | 77.31 (61.10–93.51) |  | 1322.49 (863.33–1781.64) | 419.27 (321.94–516.61) |  | 43.53 (39.02–48.03) | 13.57 (11.63–15.51) |
|  | 2038 | 244.03 (157.15–330.92) | 77.33 (60.33–94.34) |  | 1328.00 (821.02–1834.98) | 419.46 (317.26–521.66) |  | 43.84 (39.23–48.45) | 13.65 (11.66–15.65) |
|  | 2039 | 244.76 (149.50–340.02) | 77.36 (59.58–95.13) |  | 1333.51 (777.14–1889.89) | 419.61 (312.70–526.51) |  | 44.15 (39.43–48.87) | 13.74 (11.70–15.78) |
|  | 2040 | 245.48 (141.60–349.37) | 77.37 (58.85–95.89) |  | 1339.03 (731.73–1946.33) | 419.72 (308.28–531.15) |  | 44.47 (39.64–49.29) | 13.82 (11.73–15.91) |
| EU | 2024 | 140.36 (136.51–144.21) | 37.78 (37.24–38.32) |  | 977.09 (965.71–988.47) | 231.37 (229.20–233.53) |  | 29.32 (28.86–29.79) | 6.87 (6.77–6.97) |
|  | 2025 | 143.28 (137.30–149.26) | 37.88 (37.23–38.53) |  | 984.11 (967.31–1000.91) | 232.60 (229.53–235.67) |  | 29.42 (28.89–29.96) | 6.89 (6.77–7.00) |
|  | 2026 | 145.32 (137.09–153.55) | 37.98 (37.24–38.72) |  | 988.81 (967.19–1010.43) | 233.28 (229.44–237.13) |  | 29.53 (28.93–30.13) | 6.91 (6.78–7.03) |
|  | 2027 | 146.42 (135.98–156.86) | 38.07 (37.25–38.90) |  | 992.06 (966.48–1017.64) | 233.60 (229.16–238.04) |  | 29.63 (28.97–30.29) | 6.92 (6.78–7.06) |
|  | 2028 | 146.65 (134.16–159.13) | 38.17 (37.28–39.07) |  | 994.79 (966.09–1023.50) | 233.79 (228.93–238.65) |  | 29.73 (29.02–30.44) | 6.94 (6.79–7.09) |
|  | 2029 | 146.18 (131.90–160.47) | 38.27 (37.31–39.24) |  | 997.69 (966.51–1028.86) | 234.06 (228.92–239.21) |  | 29.83 (29.07–30.59) | 6.96 (6.80–7.12) |
|  | 2030 | 145.26 (129.45–161.07) | 38.37 (37.34–39.40) |  | 1001.07 (967.85–1034.29) | 234.54 (229.18–239.89) |  | 29.93 (29.13–30.74) | 6.97 (6.80–7.15) |
|  | 2031 | 144.11 (127.04–161.19) | 38.47 (37.38–39.56) |  | 1004.99 (969.99–1040.00) | 235.25 (229.72–240.78) |  | 30.03 (29.18–30.88) | 6.99 (6.81–7.17) |
|  | 2032 | 142.96 (124.85–161.08) | 38.57 (37.42–39.71) |  | 1009.32 (972.65–1045.99) | 236.15 (230.46–241.84) |  | 30.13 (29.24–31.03) | 7.01 (6.82–7.20) |
|  | 2033 | 141.96 (123.00–160.92) | 38.66 (37.46–39.86) |  | 1013.85 (975.57–1052.13) | 237.16 (231.28–243.03) |  | 30.24 (29.31–31.17) | 7.03 (6.83–7.22) |
|  | 2034 | 141.22 (121.54–160.89) | 38.76 (37.51–40.01) |  | 1018.41 (978.54–1058.28) | 238.17 (232.10–244.24) |  | 30.34 (29.37–31.31) | 7.04 (6.84–7.25) |
|  | 2035 | 140.77 (120.48–161.06) | 38.86 (37.56–40.16) |  | 1022.90 (981.45–1064.35) | 239.12 (232.83–245.41) |  | 30.44 (29.43–31.44) | 7.06 (6.85–7.27) |
|  | 2036 | 140.60 (119.75–161.45) | 38.96 (37.61–40.31) |  | 1027.28 (984.28–1070.27) | 239.98 (233.46–246.51) |  | 30.54 (29.50–31.58) | 7.08 (6.86–7.30) |
|  | 2037 | 140.68 (119.30–162.06) | 39.06 (37.66–40.45) |  | 1031.55 (987.04–1076.06) | 240.76 (234.01–247.51) |  | 30.64 (29.57–31.72) | 7.09 (6.86–7.32) |
|  | 2038 | 140.93 (119.03–162.83) | 39.16 (37.71–40.60) |  | 1035.76 (989.79–1081.73) | 241.47 (234.50–248.43) |  | 30.74 (29.64–31.85) | 7.11 (6.87–7.35) |
|  | 2039 | 141.27 (118.85–163.70) | 39.25 (37.77–40.74) |  | 1039.95 (992.56–1087.33) | 242.15 (234.98–249.32) |  | 30.85 (29.71–31.98) | 7.13 (6.89–7.37) |
|  | 2040 | 141.64 (118.67–164.61) | 39.35 (37.83–40.88) |  | 1044.14 (995.40–1092.88) | 242.85 (235.50–250.21) |  | 30.95 (29.78–32.12) | 7.14 (6.90–7.39) |
| DALYs, disability-adjusted life years; EU, European Union; CI, confidence interval; | | | | | | | | | |
