# Supplementary material for: Epidemiological trends and burden of gout in China and the European Union: a GBD 2023 and Mendelian randomization study
Source: Clin Rheumatol. 2026 May 5;45(6):3031–45. doi: 10.1007/s10067-026-08135-6 (PMC13249755; doi:10.1007/s10067-026-08135-6)
Supplement: Supplementary file 2 — Supplementary file2 (DOCX 20 KB) [file 10067_2026_8135_MOESM2_ESM.docx]

**Table S2** ARIMA model parameters and their corresponding AIC and BIC for prediction of age-standardized rate (per 100 000) of all three measures for gout for the next 17 years in China and EU

| **Location** | **Sex** | **Age-standardized incidence rate** | | | **Age-standardized prevalence rate** | | | **Age-standardized DALYs rate** | | |
| --- | --- | --- | --- | --- | --- | --- | --- | --- | --- | --- |
|  |  | Parameters | AIC | BIC | Parameters | AIC | BIC | Parameters | AIC | BIC |
| China | Male | ARIMA (1,2,1) | 15.4 | 19.6 | ARIMA (1,2,1) | 118.11 | 122.31 | ARIMA (0,2,2) | -58.63 | -54.42 |
|  | Female | ARIMA (3,1,0) | -14.04 | -8.3 | ARIMA (3,1,0) | 95.85 | 101.58 | ARIMA (2,1,0) | -90.23 | -85.93 |
| EU | Male | ARIMA (2,1,0) | 33.98 | 38.29 | ARIMA (2,1,1) | 94.24 | 101.41 | ARIMA (0,1,3) | -80.9 | -73.73 |
|  | Female | ARIMA (0,1,1) | -49.99 | -45.69 | ARIMA (2,1,0) | 6.36 | 12.1 | ARIMA (2,1,0) | -150.27 | -144.54 |

*AIC* Akaike information criterion, *BIC* Bayesian information criterion, *EU* European Union, *DALYs* Disability-adjusted life years
