# Supplementary material for: Epidemiological trends and burden of gout in China and the European Union: a GBD 2023 and Mendelian randomization study
Source: Clin Rheumatol. 2026 May 5;45(6):3031–45. doi: 10.1007/s10067-026-08135-6 (PMC13249755; doi:10.1007/s10067-026-08135-6)
Supplement: Supplementary file 3 — Supplementary file3 (DOCX 17 KB) [file 10067_2026_8135_MOESM3_ESM.docx]

Table.S3 Detailed information on instrumental variables for exposure and outcome.

| Exposure/Outcome | GWAS ID | Sample size | Year | Number of SNPs | Population | PMID |
| --- | --- | --- | --- | --- | --- | --- |
| BMI | ieu-b-40 | 681,275 | 2018 | 2,336,260 | European | 30124842 |
| Gout | ieu-a-1054 | 69,374 | 2013 | 2,450,548 | European | 23263486 |
