# Supplementary material for: Epidemiological trends and burden of gout in China and the European Union: a GBD 2023 and Mendelian randomization study
Source: Clin Rheumatol. 2026 May 5;45(6):3031–45. doi: 10.1007/s10067-026-08135-6 (PMC13249755; doi:10.1007/s10067-026-08135-6)
Supplement: Supplementary file 5 — Supplementary file5 (DOCX 17 KB) [file 10067_2026_8135_MOESM5_ESM.docx]

|  |  |  |  |  |  |  |  |
| --- | --- | --- | --- | --- | --- | --- | --- |
| Table S5 MR heterogeneity | | | | | | | |
| id.exposure | id.outcome | outcome | exposure | method | Q | Q_df | Q_pval |
|  |  |  |  |  |  |  |  |
| BMI | Gout | Gout | BMI | MR Egger | 192.8464961 | 204 | 0.70174024 |
| BMI | Gout | Gout | BMI | Inverse variance weighted | 192.8594049 | 205 | 0.718619918 |
