# Supplementary material for: Epidemiological trends and burden of gout in China and the European Union: a GBD 2023 and Mendelian randomization study
Source: Clin Rheumatol. 2026 May 5;45(6):3031–45. doi: 10.1007/s10067-026-08135-6 (PMC13249755; doi:10.1007/s10067-026-08135-6)
Supplement: Supplementary file 6 — Supplementary file6 (DOCX 16 KB) [file 10067_2026_8135_MOESM6_ESM.docx]

| Table S6. MR steiger test | | | | | | | |
| --- | --- | --- | --- | --- | --- | --- | --- |
| id.exposure | id.outcome | exposure | outcome | snp_r2.exposure | snp_r2.outcome | correct_causal_direction | steiger_pval |
|  |  |  |  |  |  |  |  |
| BMI | Gout | BMI | Gout | 0.017108915 | 0.00316173 | TRUE | 2.00E-79 |
