# Supplementary material for: Epidemiological trends and burden of gout in China and the European Union: a GBD 2023 and Mendelian randomization study
Source: Clin Rheumatol. 2026 May 5;45(6):3031–45. doi: 10.1007/s10067-026-08135-6 (PMC13249755; doi:10.1007/s10067-026-08135-6)
Supplement: Supplementary file 7 — Supplementary file7 (DOCX 16 KB) [file 10067_2026_8135_MOESM7_ESM.docx]

| Table.S7 MR pleiotropy | | | | | | |
| --- | --- | --- | --- | --- | --- | --- |
| id.exposure | id.outcome | outcome | exposure | Egger intercept | se | pval |
|  |  |  |  |  |  |  |
| BMI | Gout | Gout | BMI | 0.001107808 | 0.00975037 | 0.909653148 |
